# Proximity effect in [Nb(1.5 nm)/Fe(*x*)]_10_/Nb(50 nm) superconductor/ferromagnet heterostructures

**DOI:** 10.3762/bjnano.11.109

**Published:** 2020-08-21

**Authors:** Yury Khaydukov, Sabine Pütter, Laura Guasco, Roman Morari, Gideok Kim, Thomas Keller, Anatolie Sidorenko, Bernhard Keimer

**Affiliations:** 1 Max-Planck-Institut für Festkörperforschung, Heisenbergstraße 1, D-70569 Stuttgart, Germany; 2 Max Planck Society Outstation at the Heinz Maier-Leibnitz Zentrum (MLZ), D-85748 Garching, Germany; 3 Skobeltsyn Institute of Nuclear Physics, Moscow State University, Moscow 119991, Russia; 4 Forschungszentrum Jülich GmbH, Jülich Centre for Neutron Science (JCNS) at Heinz Maier-Leibnitz Zentrum (MLZ), Lichtenbergstr. 1, D-85747 Garching, Germany; 5 Institute of Electronic Engineering and Nanotechnologies ASM, MD2028 Kishinev, Moldova

**Keywords:** ferromagnet, iron (Fe), mixed state, neutron reflectometry, niobium (Nb), proximity effects, superconductor

## Abstract

We have investigated the structural, magnetic and superconduction properties of [Nb(1.5 nm)/Fe(*x*)]_10_ superlattices deposited on a thick Nb(50 nm) layer. Our investigation showed that the Nb(50 nm) layer grows epitaxially at 800 °C on the Al_2_O_3_(1−102) substrate. Samples grown at this condition possess a high residual resistivity ratio of 15–20. By using neutron reflectometry we show that Fe/Nb superlattices with *x <* 4 nm form a depth-modulated FeNb alloy with concentration of iron varying between 60% and 90%. This alloy has weak ferromagnetic properties. The proximity of this weak ferromagnetic layer to a thick superconductor leads to an intermediate phase that is characterized by a suppressed but still finite resistance of structure in a temperature interval of about 1 K below the superconducting transition of thick Nb. By increasing the thickness of the Fe layer to *x* = 4 nm the intermediate phase disappears. We attribute the intermediate state to proximity induced non-homogeneous superconductivity in the structure.

## Introduction

Superconductor(S)/ferromagnet(F) heterostructures are intensively studied systems, which are interesting for fundamental physics due to a big number of predicted and detected phenomena such as the appearance of non-uniform superconducting states (see reviews [1–3]). Among these phenomena are π–Josephson junctions [4–7] with a π-phase difference of superconducting correlations between two neighboring interfaces, long-range triplet superconductivity [8–16] generated in S/F systems with a non-collinear (NC) magnetic configuration of the F system, and re-entrant superconductivity as evidence of nonuniform LOFF-states [17–20]. Apart from the interest in basic science, the proximity effect in S/F structures has great technological importance for the creation of spintronics devices, where the transport properties of the structure are controlled via the manipulation of the magnetic order in the F subsystem [21–26].

One possible way to exert such a control is via interaction of superconductivity and interlayer exchange coupling (IEC) of F layers through a normal metal (NM) spacer. The IEC in a F/N/F system can be tuned by varying the thickness of the N spacer to organize antiparallel (AP), parallel (P) or non-collinearly aligned F layers [27]. Also, the presence of superconducting correlations in the same F/N/F system would favor AP alignment for singlet pairing or a NC configuration to generate a long-range triplet condensate. To the best of our knowledge the interaction of singlet superconductivity and exchange coupling was first considered theoretically in [28–29] and different magnetic re-ordering processes, such as the transition from parallel to antiparallel alignment [29] or the suppression of RKKY interaction below *T*_C_ were calculated [28]. Experimentally the interaction of exchange coupling can be studied by integral magnetometric methods such as SQUID magnetometry [30] or depth-resolved techniques such as polarized neutron reflectometry (PNR) [31].

One potentially interesting system for studying the interaction between superconductivity and IEC is the Fe/Nb system. Proximity effects in Fe/Nb systems were extensively studied before [32–36]. The antiferromagnetic coupling of Fe layers through a Nb(*y*) spacer with *y* = (1.3 + 0.9 × *n*) nm (*n* = 0, 1, 2) was found in [37–38] by means of PNR. In the following work of the same group [39] the modification of IEC by hydrogen uptake was reported. An advantage of niobium as N spacer is that it is the superconducting material with the highest bulk *T*_C_ = 9.3 K among all elemental superconductors. However, the thickest Nb spacer layer where AP alignment is still possible, *y* ≈ 3 nm, is still two times smaller than the minimum thickness 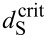 ≈ 6–8 nm of thin Nb films in which superconductivity appears [19–20]. In order to provide superconducting correlations in a Fe/Nb superlattice (SL) we propose to deposit the Fe/Nb SL on top of a thick Nb(40–50 nm) layer. This thick superconducting layer will act as a reservoir of superconducting pairs, which will be transferred to the SL using the proximity effect. The aim of this work is the study of structural, magnetic and superconducting properties of such S/F heterostructures.

## Experimental

### Growth conditions and techniques

Samples of the nominal structure Pt(3 nm)/[Nb(1.5 nm)/Fe(*x*)]_10_/Nb(50 nm) were prepared on Al_2_O_3_(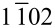) substrates using a DCA M600 MBE system with a base pressure of 10^−10^ mbar. Before deposition, the substrates were cleaned from organic contaminations with ethanol and isopropanol ex situ and heated at 1000 °C in ultra high vacuum for 2–3 h. A 50 nm thick Nb layer was deposited at a typical rate of 0.6 Å/s and a substrate temperature of *T*_Nb_ = 800 °C for samples s1 to s5 and at *T*_Nb_ = 33 °C for sample s6. Subsequently, the substrate temperature was decreased to *T*_SL_ = 30–100 °C (see below Table 1) and a periodic structure [Nb(1.5 nm)/Fe(*x*)]_10_ was deposited starting with the iron layer. The growth rates for both elements in the periodic structure were about 0.1 Å/s. On top, a 3 nm Pt cap layer was grown at about 0.3 Å/s at room temperature to protect the sample against oxidation. Fe was deposited by thermal evaporation from an effusion cell while Nb and Pt were grown by electron beam evaporation. Reflection high-energy electron diffraction (RHEED) was measured in situ during deposition to trace the structure of the atomic layer being deposited. For the RHEED experiment, an electron beam of 15 keV energy was directed along the 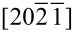 azimuth of the sapphire substrate.

In order to check the crystal structure and the quality of the epitaxial growth, X-ray diffraction measurements were performed using a θ–2θ diffractometer. The diffractometer operates at the wavelength of λ = 1.54 Å and is equipped with a DECTRIS line detector, which allows for simultaneous measurement of both specular and off-specular reflections.

The polarized neutron reflectometry (PNR) experiments were conducted on the angle-dispersive reflectometer NREX (λ = 4.28 Å) at the research reactor FRM-II (Garching, Germany). During the experiments we applied a magnetic field in-plane and normal to the sample plane. Data were fitted to models using the exact solutions of the Schrödinger equation as described in our prior works [13,40–41].

**Figure 1:**
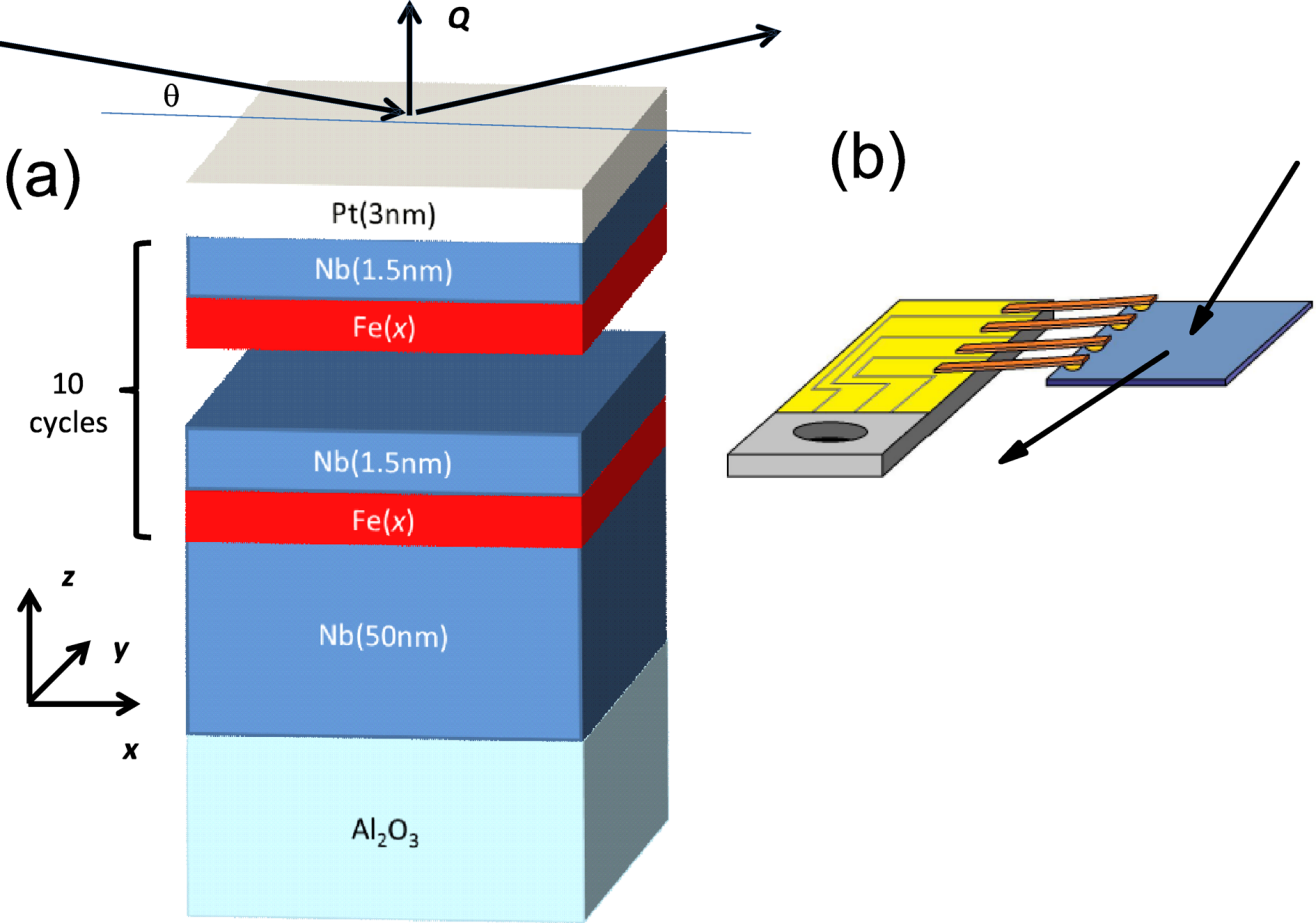
(a) Sketch of the experiment for structure and reflectometric measurements and (b) setup for the transport measurements. Black arrows show the direction of the neutron beam.

For the transport experiment we used the device depicted in Figure 1b. The device consists of four metallic springs touching the surface of the sample. The tension of the springs is sufficiently high to ensure good contact with the sample surface and to measure the resistivity using a standard four-point contact method. The setup is designed to enable simultaneous PNR and transport experiments, though in this work we used it ex situ. For the measurements we used an ac current with an amplitude of 100–200 μA. In the experiment we measured the resistance of the samples 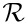 as a function of the temperature *T* and the magnetic field *H*, which was applied parallel to the sample surface. Before every *H* scan we waited 10–15 min to stabilize the temperature. From the transport measurements we derived the residual resistivity ratio RRR = 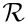
(300K)/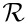
(10K), the superconducting transition temperature *T*_C_ and its width Δ*T*_C_. The latter two parameters were defined as the center and the width of derivative d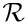/d*T*, respectively.

### Reflectometry data analysis

To study the quality of layers and interfaces in a layered structure reflectometry techniques (X-ray or neutron) can be used. Using these methods, a reflectivity curve *R*(*Q*) is measured as a function of momentum transfer *Q* = 4πsin(θ)/λ. In the kinematical approximation the reflectivity is proportional to the square of the Fourier transform of dρ(*z*)/d*z*, where ρ(*z*) is the depth profile of the scattering length density (SLD). The SLD is defined as the product of the averaged scattering length 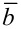 and the density *N*. For a periodic structure with period *D* repeated *n* times one can write a simple expression for the reflectivity [42]:

[1]
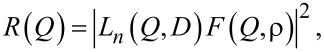


where *L_n_*(*Q*,*D*) = (1 − *e^inQD^*)/(1 − *e^iQD^*) is the Laue function and *F*(*Q*,ρ) is the structure factor of the unit cell. The latter can be written for a Fe(*x*)/Nb(*y*) periodic bilayer as

[2]
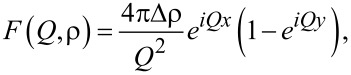


where Δρ = (ρ_Fe_ − ρ_Nb_) is the contrast between the SLDs of Fe and Nb. Thus, from Equation 1 and Equation 2 it follows that reflectometry measures the contrast between SLDs of neighboring layers. Using Equation 1 and Equation 2 we can write for the reflectivity *R*_1_ at the first Bragg peak *Q*_1_ = 2π/*D*:

[3]
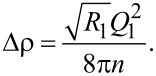


Thus, the Bragg analysis allows us to determine the contrast between the SLDs of Fe and Nb. Interdiffusion will lead to suppression of the contrast and, hence, of *R*_1_. Assuming that the packing density *N*_av_ is the same for both layers, we may estimate the concentration of Fe in the Fe*_c_*Nb*_1−c_* alloy as

[4]
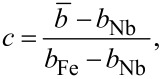


where 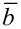 = ρ/*N*_av_ is the averaged coherent scattering length of the corresponding layer, and *b*_Fe_ and *b*_Nb_ are the coherent scattering lengths of Fe and Nb. The X-ray SLDs of Fe and Nb differ only by a few percent, which makes the X-ray contrast very small even without interdiffusion. For neutrons, in contrast, the SLDs of Fe and Nb, ρ_Fe_ = 8 × 10^−4^ nm^−2^ and ρ_Nb_ = 3.9 × 10^−4^ nm^−2^, differ by a factor of two, which makes neutron reflectometry a better choice to study diffusion in periodic Fe/Nb structures. Another advantage of neutron reflectometry is its sensitivity to the magnetic depth profile. The total SLD for spin-up(+) and spin-down(−) neutrons can be written as ρ^±^(*z*) = ρ_0_(*z*) ± ρ_m_(*z*), where ρ_0_ and ρ_m_ are the nuclear and magnetic SLDs. The latter is proportional to the magnetization of a layer. Thus in addition to the chemical we can study magnetic depth profiles using PNR.

## Results

### Structural study

#### Growth analysis with RHEED

**Figure 2:**
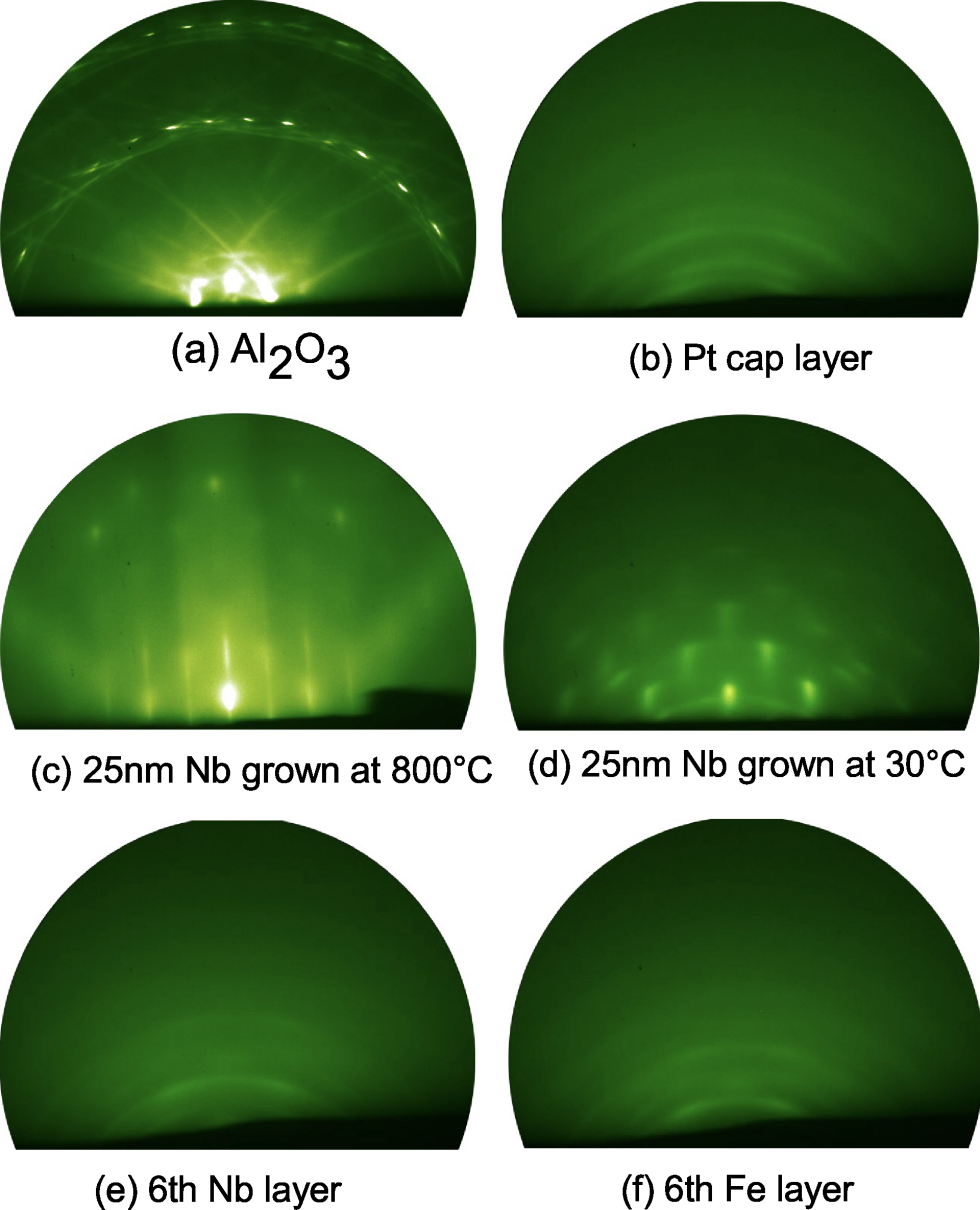
RHEED patterns of (a) the Al_2_O_3_(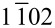) substrate, (b) the Pt cap layer, and the Nb buffer layer grown at (c) 800 °C and (d) 30 °C. (e, f) Growth stages of sample s6: (e) the 6th Fe layer, (f) the 6th Nb layer.

The RHEED pattern of the Al_2_O_3_(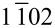) substrate (Figure 2a) reveals a crystalline structure with Laue rings and Kikuchi lines indicating a smooth and ordered surface. Nb deposition at 800 °C results in a streaky pattern and a Laue ring (Figure 2c) revealing epitaxial growth in agreement with previous results [43–46]. In particular, the epitaxial Nb growth of (100) orientation on Al_2_O_3_(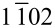) substrates was reported in [46]. The peculiarity of this growth, also seen in our samples, is an angle of approx. 3° between the above mentioned planes of Nb and Al_2_O_3_(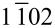). At *T*_Nb_ = 30 °C a transmission pattern (i.e., a regular arrangement of spots) and rings are visible in the RHEED pattern of the Nb layer, which indicate island growth and polycrystallinity (Figure 2d).

Subsequently, the Fe/Nb multilayers were grown on the 800 °C Nb buffer. The corresponding RHEED patterns exhibit amorphous growth, i.e., blurred screens (not shown). Increasing the Fe film thickness from 2 to 4 nm improves the film quality. The Fe layer becomes polycrystalline while the Nb layer remains amorphous. In contrast, for sample s6, which was grown on the 30 °C Nb buffer, both layers reveal polycrystallinity with a certain texture (Figure 2e,f). Finally, the Pt cap is always polycrystalline (Figure 2b).

#### X-ray diffraction

**Figure 3:**
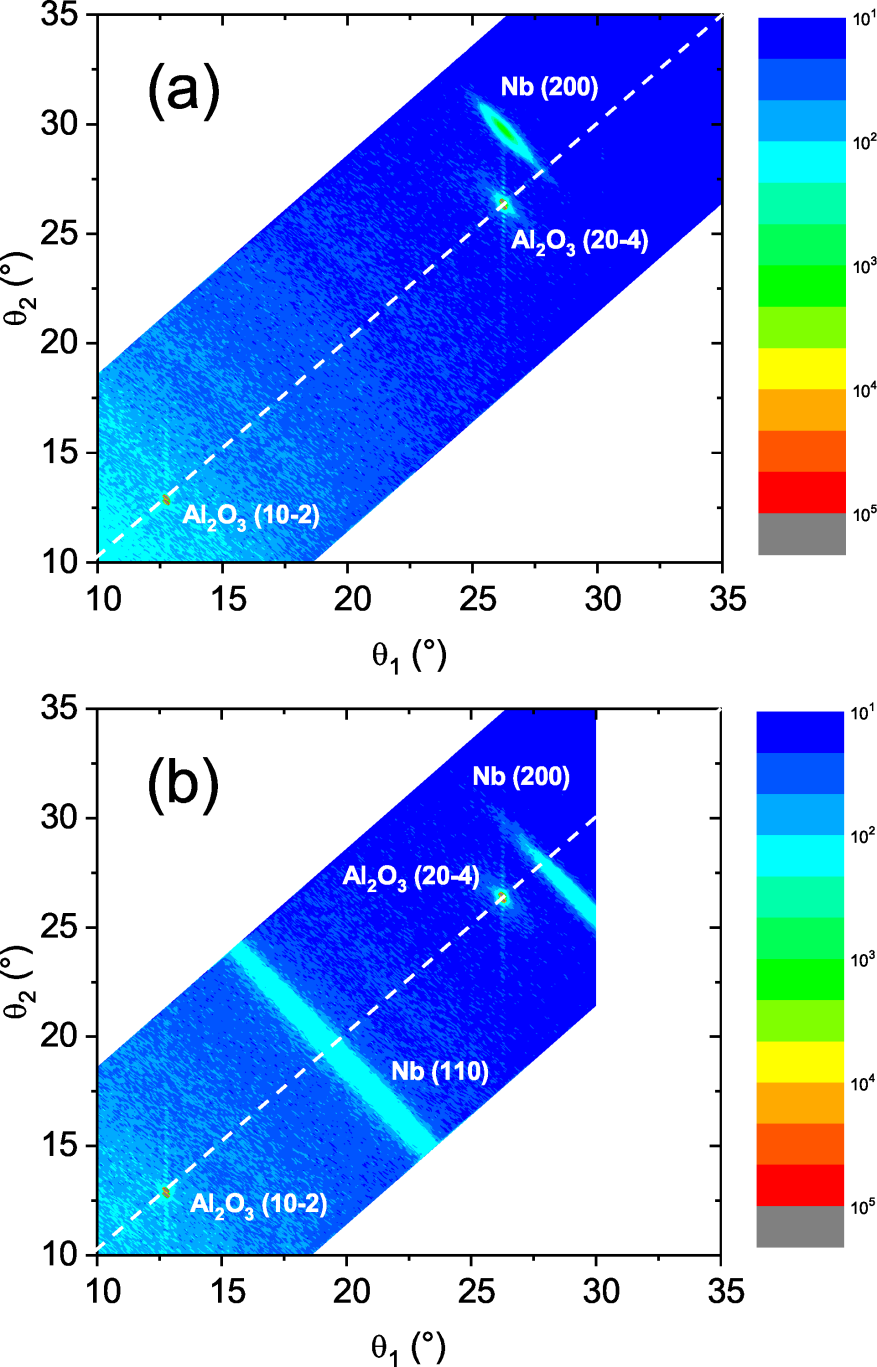
X-ray diffraction patterns of (a) sample s3 and (b) sample s6. Dashed tilted lines show the direction of specular reflection. The color bars show the logarithmic intensity scale.

**Figure 4:**
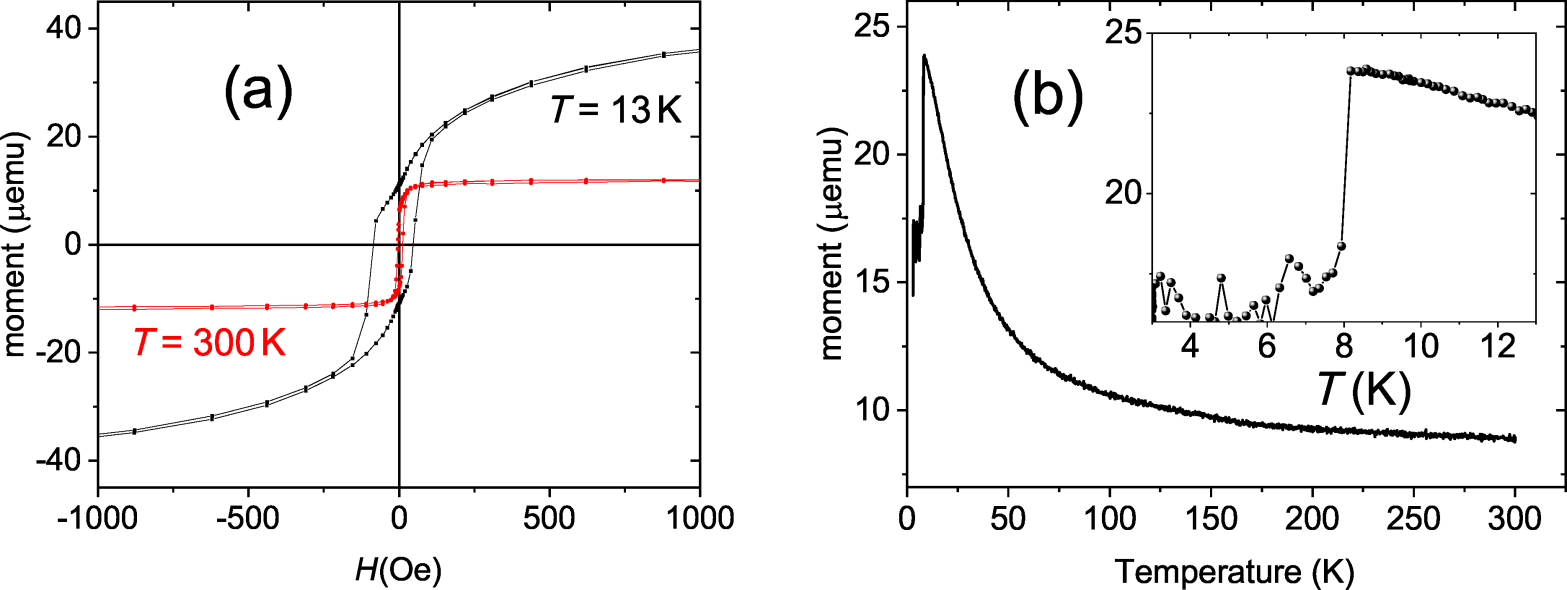
SQUID data of the s3 sample. (a) Hysteresis loop measured at *T* = 300 K (red) and *T* = 13 K (black). (b) Temperature dependence of the magnetic moment measured at *H* = 250 Oe. The inset shows the data in the vicinity of the superconducting transition.

Figure 3a shows the diffraction pattern measured on sample s3. Together with two reflections from the substrate we observed a Nb(200) peak at 2θ = 55°with mosaicity of the same order as the substrate peak. In agreement with the observation by RHEED (Figure 4b) we observed that the Nb(200) peak is tilted off-specular by a few degrees, which is a well-known feature of Nb growth on Al_2_O_3_(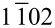) substrates [45–46]. Similar patterns were measured for all samples, except for sample s6, which was grown at room temperature. For this sample we measured a typical polycrystalline pattern with coexisting Nb(100) and Nb(110) phases (Figure 3b).

### Magnetic properties

#### SQUID measurements

Figure 4a shows hysteresis loops measured on sample s3 at *T* = 300 K and *T* = 13 K. At room temperature the sample saturates to a magnetic moment *m*_sat_ = 12 μemu above a saturation field of only *H*_sat_ = 50 Oe. At 13 K the saturation moment increases to *m*_sat_ = 40 μemu and a field above *H*_sat_ ≈ 2 kOe is needed to saturate the magnetic moment of the sample. The temperature dependence of the magnetic moment at *H* = 250 Oe (Figure 4b) shows that the moment is constant down to *T* ≈ 100 K, and grows upon further cooling to *T* = 8.2 K. Below this temperature a decrease of the magnetic moment due to the Meissner effect is observed.

#### Polarized neutron reflectometry

**Figure 5:**
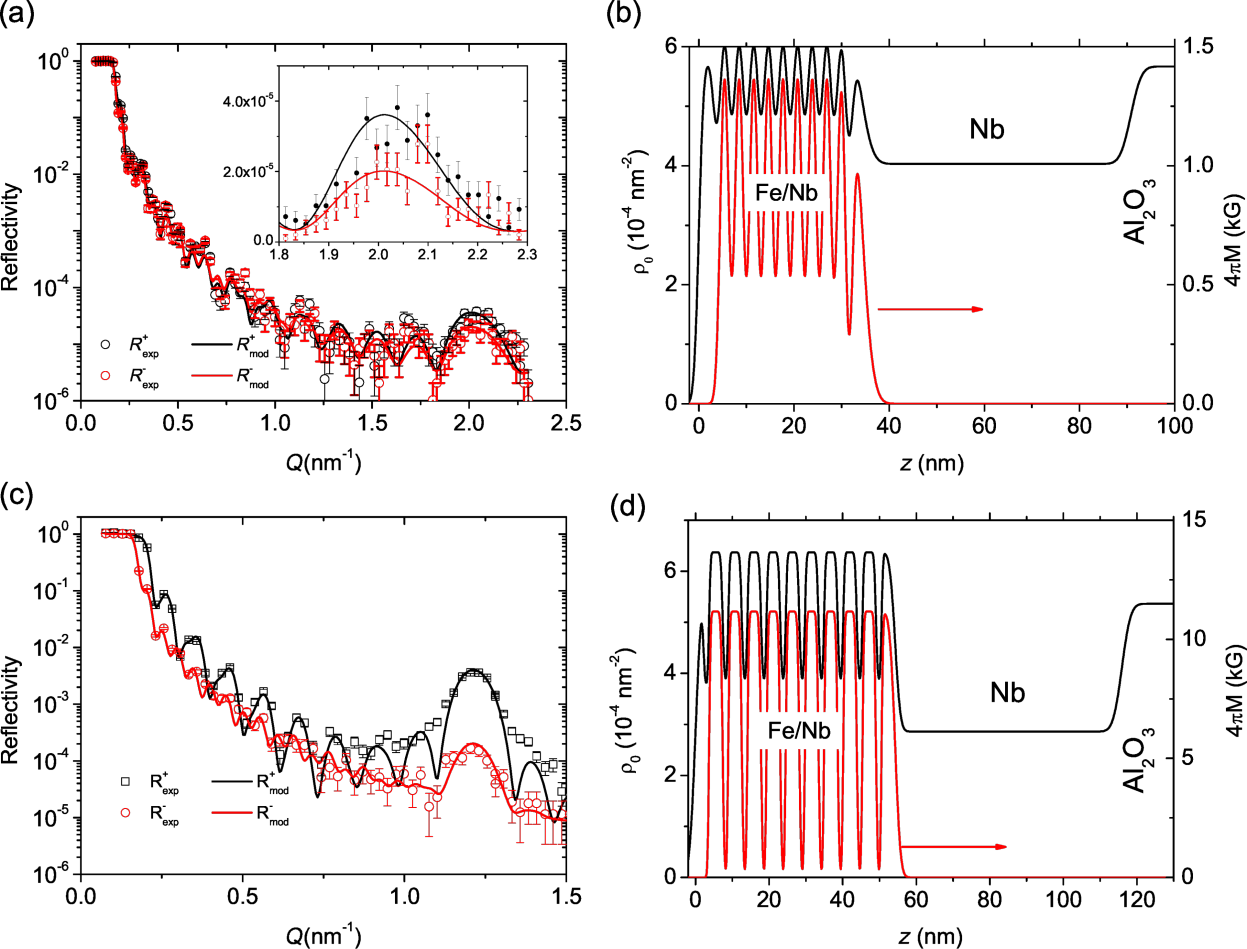
(a) Experimental (dots) and model (lines) reflectivity curves measured on sample s3 at *T* = 13 K and *H* = 4.5 kOe. (b) The depth profiles of SLD and magnetization for the same sample. (c) Reflectivity curves for sample s4 measured at *T* = 300 K and *H* = 5 Oe. (d) Corresponding depth profile of SLD and magnetization for sample s4.

Figure 5a shows reflectivity curves measured on sample s3 at a temperature of *T* = 13 K in a magnetic field of *H* = 4.5 kOe. The curves are characterized by the total external reflection plateau, interference oscillations and the first Bragg peak at *Q*_1_ ≈ 2.1 nm^−1^. The intensity of the Bragg peak *R*(*Q*_1_) ≡ *R*_1_ ≈ 4 × 10^−5^ is one order of magnitude lower than calculated for the nominal SLDs, indicating high interdiffusion of Fe and Nb. Despite this high interdiffusion we observed a statistically significant difference of Bragg intensities for spin-up and spin-down neutrons (see inset in Figure 5a), which suggests the presence of magnetism in the periodic structure. A similar picture was also observed for the samples s1 and s2, which shows that the interdiffusion does not depend strongly on the deposition temperature *T*_SL_. We fitted experimental curves to models with varying SLD, thickness, and rms roughness of all layers and varying magnetization of the Fe layer. The resulting depth profiles ρ_0_(*z*) and *M*(*z*) are shown in Figure 5b. According to our model the SLD in the center of the Fe and Nb layers is ρ_Fe_ = 6.0(2) × 10^−4^ nm^−2^ and ρ_Nb_ = 5.0(2) × 10^−4^ nm^−2^, respectively. Using Equation 4 for *N*_av_ = (*N*_Fe_ + *N*_Nb_)/2 we can estimate the concentration of iron atoms in the nominal Fe and Nb layers as *c* = 90% and *c* = 60%, respectively. In this estimation we used the bulk densities *N*_Fe_ = 0.085 Å^−3^, *N*_Nb_ = 0.06 Å^−3^ and the scattering lengths *b*_Fe_ = 9.45 fm and *b*_Nb_ = 7.05 fm.

Samples s4 to s6 were measured at room temperature at *H* = 4.5 kOe and analyzed in the same way. The resulting SLDs and magnetization values are shown below in Table 1. All samples except s4 show strong intermixing of Fe and Nb atoms, which resulted in a suppressed magnetization of the order of 10% of the bulk value. For sample s4 with Fe(4 nm) we observed a much stronger Bragg peak with significantly stronger difference of spin-up and spin-down channels (Figure 5c). The fit shows (Figure 5d) that these reflectivities correspond to much higher nuclear and magnetic contrast.

Since the thickness of our Nb spacer, 1.3 nm, is close to the thickness at which antiferromagnetic coupling was observed in [37–39] we thoroughly searched for antiferromagnetic coupling in our structures. We remind that such a coupling will lead to the doubling of the magnetic period comparing to the chemical one and, hence, the appearance of Bragg peaks on positions *Q*_AF_ = 1/2 *Q_n_*, where *n* is the order of the structural Bragg peak. In addition to the high-field measurements we performed PNR measurements at a low magnetic field of *H* = 5 Oe. None of our measurements revealed the appearance of additional peaks. An example is given in Figure 5c for sample s4 measured at room temperature in *H* = 5 Oe.

### Transport measurements

**Figure 6:**
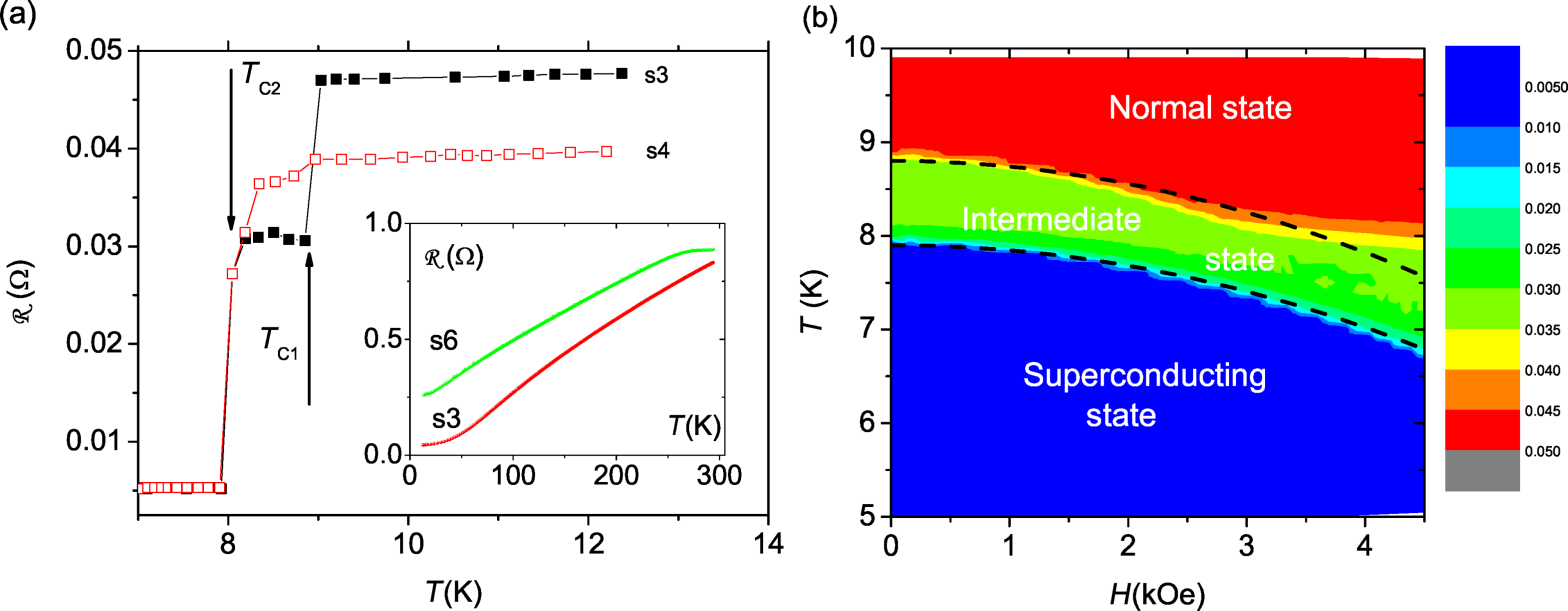
(a) 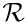
(*T*) for the samples s4 (black) and s3 (red) in the vicinity of the superconducting transition measured in zero magnetic field. The inset shows the resistance of samples s3 and s6 between room temperature and 10 K in *H* = 4.5 kOe. (b) The *H*–*T* dependence of resistance of the sample s3. The color bar depicts resistance in ohms. The bottom dashed line show the dependence *T_c_*_2_(*H*) = *T_c_*_2_(0) [1 − (*H*/*H_c_*_2_(0))^2^] with *H_c_*_2_(0) = 12 kOe. The upper dashed line is shifted 0.9 K up from the bottom one to show borders of the intermediate state.

The inset of Figure 6a shows the resistance 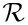
(*T*) of samples s3 and s6 measured during cooling from room temperature to 10 K in magnetic field of *H* = 4.5 kOe. For s3 we measured RRR = 18.6, a value which is typical for MBE-prepared S/F structures in the epitaxial regime of growth [47–48]. Similar values of RRR from 16 to 20 were obtained for all samples except RRR = 3.4 for s6, which was deposited at room temperature and has polycrystalline quality (see below Table 1).

Figure 6a shows the 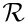
(*T*) curves for the s3 and s4 measured in zero magnetic field in the vicinity of the superconducting transition. For sample s3 we observed a 40% drop of resistance below *T_c_*_1_ = 8.9 K. A similar drop was observed for all samples, except for s4, for which the initial drop was only 3%. Finally, below *T_c_*_2_ ≈ 8 K the resistance falls to zero (down to the accuracy of the setup-related background resistance of 5 mΩ) for all samples, evidencing the superconducting transition. The second transition coincides with the transition seen by SQUID (inset in Figure 4b).

Figure 6b shows the 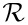
(*H*,*T*) phase diagram for sample s3. The superconducting transition can be well described by the expression *T_c_*_2_(*H*) = *T_c_*_2_(0) [1 − (*H*/*H_c_*_2_(0))^2^] with *H_c_*_2_(0) = 12 kOe. This expression can be re-written in the well known form for 2D superconductors: 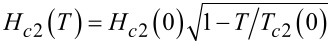. From *H_c_*_2_(0) we can estimate the superconducting correlation length ξ_S_ = 12 nm. We note that 2D superconductivity was reported earlier for various structures with Nb layer thicknesses of 100 nm or less [5,33,35–36,41].

## Discussion

In this work we studied the structural, magnetic and superconduction properties of [Fe(*x*)/Nb(1.5 nm)]_10_ superlattices on top of a thick Nb(50 nm) layer. The main characteristics are summarized in Table 1. Our investigation has shown that the Nb layer grows epitaxially on the Al_2_O_3_(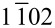) substrate in the (100) direction at a substrate temperature during deposition of *T*_Nb_ = 800 °C. This result agrees with [45]. Furthermore the samples grown at this temperature show high residual resistivity ratios of 15–20. The sample deposited at room temperature, in contrast, possesses a polycrystalline structure of the Nb(50 nm) layer with a mixture of (100) and (110) phases and a rather low RRR of 3.4, which is attributed to enhanced scattering of conduction electrons at the grain boundaries.

**Table 1:** Main characteristics of the prepared samples. Here *x* is the thickness of the Fe layers, the column “XRD” gives the corresponding Miller indices of the peaks observed in the experiment, *T*_Nb_ and *T*_SL_ are the temperatures of deposition of the thick Nb layer and the periodic structure, respectively, ρ_Fe_ and ρ_Nb_ are, respectively, the nuclear SLD at the center of the nominal Fe and Nb layers, and *T_c_*_1_ and *T_c_*_2_ are, respectively, the upper and lower transition temperature.

ID	*x* (Å)	XRD	*T*_Nb_ (°C)	*T*_SL_ (°C)	ρ_Fe_/ρ_Nb_	*M*_Fe_ (kG)	RRR	*T_c_*_1_ (K)	*T_c_*_2_ (K)

s1	15.4(1)	Nb(200)	800	100	6.1/5.1	1(1)	18.1	8.9(3)	8.1(3)
s2	15.6(4)	n/m	800	66	5.7/4.7	1.8(8)	16.9	8.9(1)	8.0(2)
s3	15.1(1)	Nb(200)	800	30	6.0/4.9	2(1)	18.6	8.8(2)	8.1(2)
s4	36.7(3)	Nb(200)	800	30	6.6/4.0	11.2(4)	19.6	8.8(2)	8.0(1)
s5	26.7(4)	Nb(200)	800	30	6.1/3.9	2(1)	16.3	8.9(1)	7.9(2)
s6	20.1(5)	Nb(110), Nb(200)	30	30	5.0/4.6	2.3(5)	3.4	9.1(1)	8.3(1)

Neutron reflectometry has shown that Fe/Nb superlattices with *x* ≤ 2.5 nm form a depth-modulated FeNb alloy with the concentration of iron varying within the superlattice unit cell between 60% and 90%. Based on the SQUID data (Figure 4), we can attribute the magnetic signal at room temperature to the iron-rich Fe_0.9_Nb_0.1_ alloy, while the signal below *T_m_* ≈ 100 K originates from Fe_0.6_Nb_0.4_. Although the thickness of our Nb spacer, 1.3 nm, is close to the values in [37–39] we did not observe any antiferromagnetic coupling, neither at room temperature nor in low-temperature measurements. The reason of this disagreement may originate from the amorphous Nb spacers.

The proximity of this depth-modulated and weakly magnetic layer to a thick superconductor causes the appearance of an intermediate phase between the normal state (*T > T_c_*_1_) with nonzero resistance and the superconducting state (*T < T_c_*_2_) with zero resistance. This state is characterized by a ≈50% suppressed resistance and the absence of the Meissner effect. Similar steps were already observed in several works [19,30,49–50]. Up to now there exists no explanation of such steps except for cases related to different imperfections of the sample (e.g., thickness gradient, crystal inhomogeneity, or oxide at S/F interface) [19,30,49–50]. However, a detailed study of our systems using various structural methods allows us to exclude these cases. To discuss this effect we first need to pay attention to the peculiarities of our transport experiment. Since the contacts are attached to the surface, electrical current will tend to flow along the surface penetrating only to a certain depth λ. To calculate λ one needs to know the resistivity of all layers in *z* direction as well as the interfaces resistances. This will require additional experiments on simpler systems and/or with current applied normal to the surface. However, the following arguments allow us to say that the value of λ is comparable to the thickness of the entire structure, that is, the current flows not only along the SL but also through the thick niobium layer or at least through its upper part. First of all, we note that a stand-alone Fe/Nb SL structure itself hardly can be a superconductor due to the absence of a clean and oriented Nb phase. Secondly, we draw attention to the difference between the transport properties of samples s5 and s6. Lowering of the deposition temperature of thick niobium *T*_Nb_ led to polycrystalline growth of the thick Nb layer of sample s6 and a strong decrease of its RRR (Table 1). Such a sensitivity of the RRR on the crystal structure of thick Nb indicates that the transport experiment is sensitive to the thick niobium layer, i.e., the current does flow through it. If this is the case, then the transition of the thick niobium to the superconducting state should lead to a (almost) complete loss of resistance. This is indeed observed experimentally below *T_c_*_2_ for all samples, while for samples with *x* ≤ 2.5 nm in the region of temperatures between *T_c_*_1_ and *T_c_*_2_ the loss is only partial. Taking into account all of these facts we can propose that the thick Nb layer in the intermediate state comprises superconducting and normal-state domains. This hypothesis allows us to explain the suppression of the resistance and the absence of the Meissner effect in the intermediate state. However, it requires further experimental and theoretical studies. For example covering the SL from both sides with thick superconductor layers would allow direct measurements of the proximity effect using PNR [41].

## Conclusion

We studied the structural, magnetic and superconducting properties of [Nb(1.5 nm)/Fe(*x*)]_10_ superlattices deposited on a thick Nb(50 nm) layer. Our investigation showed that a high deposition temperature of *T*_Nb_ = 800 °C results in systems of high structural quality with an epitaxial Nb(50 nm) layer and high residual resistivity ratios of 15–20. By using neutron reflectometry we have shown that Fe/Nb superlattices with *x <* 4 nm form a depth-modulated FeNb alloy with the concentration of iron varying between 60% and 90%. This alloy has properties of a weak ferromagnet with a Curie temperature of *T*_m_ ≈ 100 K. The proximity of this weak F layer to a thick superconductor leads to the presence of an intermediate phase between normal and superconducting state. This phase is characterized by a suppressed resistance of the structure in the temperature range of *T* = 8–9 K below the superconducting transition of thick Nb. By increasing thickness of Fe layer to *x* = 4 nm this phase was destroyed.
